# Gender and Liver Steatosis Discriminate Different Physiological Patterns in Obese Patients Undergoing Bariatric Surgery: Obesity Center Cohort

**DOI:** 10.3390/nu15102381

**Published:** 2023-05-19

**Authors:** Rossella Donghia, Rita Schiano Di Cola, Filomena Cesaro, Andrea Vitale, Giuseppe Lippolis, Teresa Lisco, Roberta Isernia, Giovanni De Pergola, Sara De Nucci, Roberta Rinaldi, Marina Liso, Cristiano Giardiello

**Affiliations:** 1 National Institute of Gastroenterology—IRCCS “Saverio de Bellis”, 70013 Castellana Grotte, Italy; 2 Pineta Grande Hospital, 81030 Castel Volturno, Italy

**Keywords:** bariatric surgery, obesity, gender, steatosis

## Abstract

Background: Obesity is a major public health problem worldwide. Bariatric surgery can reduce body weight, and it is one of the better ways to improve metabolic disease and lifestyle. The aim of this study was to explore a new cohort of patients with obesity and evaluate the gender differences and the steatosis status within the gender group. Methods: A cohort of 250 adult obese patients with BMI ≥ 30 and age >18 years, eligible for gastric bariatric surgery at Pineta Grande Hospital, Castel Volturno (Italy) was studied. Results: The prevalence in women was higher (72.40%) than men (27.60%). Overall, results indicated many statistically significant gender differences in hematological and clinical parameters. Analysis of the subcohorts based on the severity of steatosis revealed differences of this condition between the genders. Steatosis was more prevalent in the male subcohort, but female patients revealed greater within-group differences. Conclusions: Many differences were found not only in the total cohort but also between the gender subcohorts, both in the presence and absence of steatosis. We can conclude that the pathophysiological, genetic, and hormonal patterns affecting these patients delineate different individual profiles.

## 1. Introduction

Over the last few decades, obesity has become a growing public health problem worldwide, and the related conditions differ by geographic region [1]. Italy follows the trend of the progressive increase of the prevalence of obesity, showing a rise by almost 30% in the adult obese population [2]. Obesity, traditionally defined as an excess of body fat causing prejudice to health [3], is the result of complex relationships among genetic, lifestyle, socioeconomic, and cultural factors because obese individuals tend to have a lower grade of instruction and lower income [4]. In addition, obesity may be a risk factor for the development of comorbidity conditions.

Bariatric surgery is a primary component in determining weight loss as well as costs associated with obesity. According to the guidelines of the Italian Society for Bariatric Surgery (SICOB), patients with class III or II obesity and with other comorbidities are eligible for bariatric surgery that is fully covered by the Italian National Health System (SSN) [5]. However, only 1.4% of eligible patients, on average, undergo bariatric surgery in Italy, as stated in the Italian report “Osservatorio PariSanità” [6]. The main contraindications to surgery are psychological features, indicating that a patient would not be able to cope with the impact of the procedures. Pragmatic opinion-based decisions after a multidisciplinary team assessment are currently the best solution hospitals can offer patients prior to referring them for bariatric surgery [7], because not all candidates are suitable for surgery. The most commonly performed procedures are the Roux-en-Y gastric bypass (RYGB), adjustable gastric banding (AGB) and sleeve gastrectomy (SG) [8]. All procedures can be performed laparoscopically and have a low rate of complications such as wound infection and incisional hernias [9]. Gender is an important risk factor for the development of obesity. Kappoor et al. [10] had hypothesized that factors leading to gender disparities in obesity could include socioeconomic and sociocultural status, hormone-related comorbidities, and metabolic complications. The literature on this aspect is poor and does not provide details about types of prevention and evaluation. Moreover, the majority of bariatric patients are female, nearly half of which are of peri- or post-menopausal age [11]. Men tend to develop complications from obesity more easily. Obesity induces a state of chronic, low-grade inflammation responsible for the development of the metabolic syndrome and its pathophysiological consequences, such as insulin resistance, cardiovascular disease and other nonmetabolic complications of obesity [12]. 

Steatosis is an accumulation of triacylglycerols within hepatocytes based on an unbalanced synthesis or delivery of fatty acids in the liver [13]. The main aspect involved in this gender obesity study was the prevalence of steatosis, which was more prevalent in male than in female patients, even if females account for the majority of the obesity population. The severity of steatosis has been associated with increased visceral fat mass, insulin resistance, lower adiponectin levels, and higher blood pressure [14]. Not all patients with steatosis are overweight or obese [15], and vice versa, not all obese patients have steatosis.

The aim of this article was to study, for the first time, a cohort of obese patients in Southern Italy, exploring the epidemiological and physiological differences between genders and liver conditions.

## 2. Materials and Methods

Between 1 January and 31 December 2021, 250 adult obese patients with BMI ≥ 30, and age > 18 years, male and female, eligible for gastric bariatric surgery at a medical center in southern Italy (Pineta Grande Hospital, Castel Volturno, Italy), were included in this study, but the funded investigation was conducted at National Institute of Gastroenterology—IRCCS “Saverio de Bellis”.

SARS-CoV-2 has negatively affected the number of patients undergoing bariatric surgery, despite the consequent increase in obesity [16]. Before the bariatric surgery, all patients were evaluated by a bariatric team, including internists, dietitians, and psychologists. All patients were prescribed a nutritional regime by the dietitians based on their clinical and physiological characteristics. Three different kinds of diet were assigned: Ketogenic, low-carb or balanced hypocaloric diet, consisting of a maximum daily intake of 800, 1000 and 1200 Kcal, respectively. The diets were created using WinFood 3.29 software (Medimatica Srl Unipersonale, Colonnella, Italy). The detailed bromatological compositions are indicated in Table S1.

Between the prehospitalization and the day before the surgery, there are at least 14 days, which is the minimum to record an effect of the diet on the patient’s physiological parameters.

Liver steatosis was established by abdominal ultrasound screening and was graded based on liver echogenicity: 0 (absent), 1 (minimal), 2 (mild), 3 (moderate), and 4 (severe) [17]. All participants signed an informed consent form. The study was approved by Ethical Committee Campania Nord (approval date: 26 April 2023; approval code is CECN/2092) with the ethical standards of Helsinki declaration (World Medical Association, Ferney-Voltaire, France, 2013).

### Statistical Analysis

Obese patients’ characteristics are reported as mean and standard deviation (M ± SD) for continuous variables and as frequency and percentages (%) for categories. To test the association between independent groups (Female vs. Male, and Steatosis Moderate/Severe vs. Steatosis No/Mild), chi-square or Fisher’s exact test was used for categorical variables, where necessary, while the Wilcoxon rank- sum (Mann–Whitney) test was used for continuous variables. In addition, the Wilcoxon matched-pairs signed-rank test for continuous variables was applied to evaluate variations between prehospitalization/surgery, only for variables recorded in repeated tests.

To summarize and visualize the information about prehospitalization blood parameters and to create a profile of patients by gender and steatosis, a principal component analysis (PCA) was performed. This was useful to extract information from the data matrix and reduce it to fit new groups of variables. To test the null hypothesis of no association, the two-tailed probability level was set at <0.05. Analyses were conducted with StataCorp.2021 software, Release 17, StataCorp LLC., College Station, TX, USA, while for the graphics, RStudio (“Elsbeth Geranium” Release) was used.

## 3. Results

As shown in Table 1, the total obese cohort consisted of 69 (27.6%) male and 181 (72.4%) female patients. No significant differences were observed for age (both continuous and categorical) between the gender subgroups (*p* = 0.45, and *p* = 0.12, respectively). As expected, the BMI in each group was >30, but no statistically significant difference was observed (*p* = 0.54). The prevalence of employed men was higher than of employed women, and the difference was also statistically significant (51.5% vs. 82.0%, *p* = 0.001). Regarding blood parameters, obese females had lower levels of albumin (%), ratio A/G (albumin/globulin), beta 2 (%), beta 2, glycemia, triglycerides (TG), creatinine, CPK (creatine kinase), GGT (gamma-glutamyl transferase), iron, ferritin, GPT (glutamic-pyruvic transaminase), FT3 (triiodothyronine free), RBC (red blood cell), Hgb (hemoglobin), HCT (hematocrit), MCH (mean corpuscular hemoglobin), MCHC (mean corpuscular hemoglobin Concentration), PDW (platelet distribution width), monocytes, and eosinophils, with a statistically significant *p*-value (*p* < 0.05). In contrast, males showed significantly lower levels of transferrin, TSH (thyroid stimulating hormone), PTH Intact (parathyroid hormone intact), HDL (high-density lipoprotein), platelets, PCT (procalcitonin), and ESR (red blood cells sedimentation rate). Hypertension was more prevalent in males (55.1% vs. 39.8% *p* = 0.003), and the pathological condition worsened as liver volume increased (77.4% vs. 55.9%, *p* = 0.003). Moderate/severe steatosis (85.2% vs. 66.7%, *p* = 0.006) and altered gallbladder volume showed the same trend as in the female subcohort but with statistically significant differences (*p* = 0.003, *p* = 0.006, and *p* = 0.03, respectively). 

In Table 2, the same cohort was stratified by gender but also by the presence of liver steatosis (No/Mild vs. Moderate/Severe).

**Figure 1:**
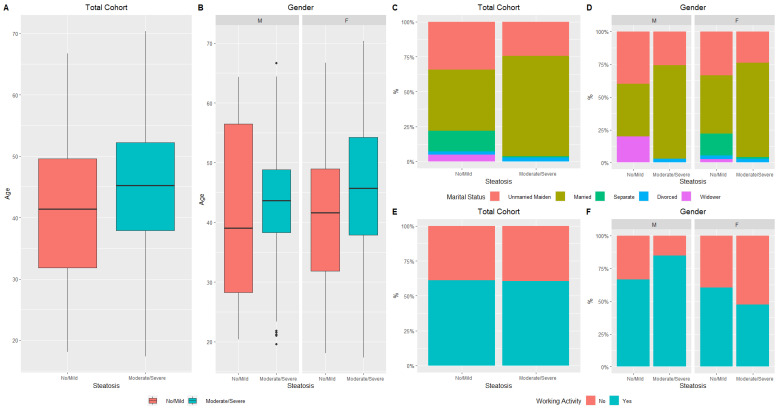
Distribution of liver steatosis (No/Mild vs. Moderate/Severe) in total cohort and gender, stratified by age (**A**,**B**), marital status (**C**,**D**), and working activity (**E**,**F**).

Steatotic patients were older, both in the total cohort and in the female subcohort (44.6 ± 11.9 vs. 40.8 ± 12.0, *p* = 0.03 and 45.5 ± 12.0 vs. 40.8 ± 11.3, *p* = 0.01) (Figure 1A,B). 

In the same way, marital status showed statistically significant differences both in the total cohort and in the female subcohort (*p* < 0.001 and *p* = 0.003, respectively) (Figure 1C,D). Regarding social status, employed male patients had a higher prevalence of steatosis than employed female patients (84.8% vs. 47.5%, *p* < 0.001) (Figure 1E,F). 

Within the category with a moderate/severe steatosis clinical status, the BMI was higher in patients in the total and female subcohorts (*p* = 0.02 and *p* = 0.008, respectively). The choice of diet and subsequent bariatric surgery reflected a prevalence of a ketogenic diet (*p* = 0.05 and *p* = 0.03) and sleeve surgery (*p* = 0.009 and *p* = 0.01) in steatosis patients, both in the total cohort and in females, as compared to patients without or with mild steatosis. In relation to the health status, hospitalization days were increased in the total and female subcohorts with moderate and severe steatosis (3.8 ± 2.9 vs. 3.3 ± 2.3 with *p* = 0.001, and 3.8 ± 3.2 vs. 3.3 ± 2.5 with *p* = 0.002, respectively). In the total cohort with moderate/severe steatosis, we found high levels of alfpha-2 (%) and alpha-2, triglycerides, GOT, GGT, ferritin, cholinesterase, GPT, PTH Intact, WBC, RBC, Hgb, HCT, neutrophils, HbA1c, and glycosylated hemoglobin, and low levels of HDL, and lymphocytes (*p* = 0.02, and *p* = 0.05 respectively). The male subcohort had high levels of alpha-1, GPT, HbA1c, and glycosylated hemoglobin, but lower levels of total and indirect fractional bilirubinemia, *p*-values <0.05. Instead, in female patients, differences were more evident between the two steatosis conditions. Patients with moderate/severe steatosis had high levels of alpha-2 (%) and alpha-2, triglycerides, ferritin, cholinesterase, GPT, PTH Intact, and WBC, *p* < 0.05. Blood group (0) was differently distributed, being most prevalent in female patients with moderate/severe steatosis, while the AB group had a lower prevalence of severe steatosis. Naturally, liver volume was increased in pathological conditions in all groups, and gallbladder stones in the total cohort had a higher prevalence in disease conditions (20.7% vs. 8.9%, *p* = 0.05). Investigation by gender of patients with no/mild steatosis highlighted that gamma (%), alpha-1, and ESR showed lower levels, as well as indirect fractional bilirubinemia, creatinine, iron, ferritin, potassium, RBC, Hgb, HCT, and MCV, as compared to the steatotic group. Instead, comparison between this disease group by gender showed that females had higher levels of alpha-2, transferrin, TSH, PTH Intact, HDL, platelets, PCT, and ESR, but lower levels of total protein, albumin, A/G ratio, beta-2, glycemia, creatinemia, CPK, GGT, iron, ferritin, FT3, RBC, Hgb, HCT, MCH, MCHC, PDW, monocytes, eosinophils. Hypertension was more prevalent in males than females (55.8% vs. 38.6%, *p* = 0.03) in moderate/severe steatosis conditions and gallbladder volume, where males manifested worse conditions (such as a contracted or scleroatrophic gallbladder). 

Table 3 shows changes in blood parameters from prehospitalization to bariatric surgery preparation after prescription of the diet to reduce weight by at least 10% of the initial weight. Both males and female showed a decrease in azotemia, creatinine, GGT, ALP, lipase, RBC, Hgb, HCT, platelets, PCT, eosinophils, and basophils, *p*-value < 0.05. Female patients also had a decrease in prothrombin time (94.8 ± 18.4 vs. 108.2 ± 20.5, *p* = 0.0005), MCV (84.9 ± 6.5 vs. 84.9 ± 6.2, *p* = 0.03), and RDW-CV (14.2 ± 1.2 vs. 14.4 ± 1.1, *p* = 0.03). Meanwhile, the diet had the effect of increasing sodium, WBC (white blood cells), and neutrophils in both genders. In particular, male patients had an increased MCV (86.9 ± 6.4 vs. 86.7 ± 4.4, *p* = 0.05), whereas females showed a decrease. Female patients also showed a decrease of I.N.R. (1.0 ± 0.1 vs. 1.0 ± 0.2, *p* = 0.0003). 

**Figure 2:**
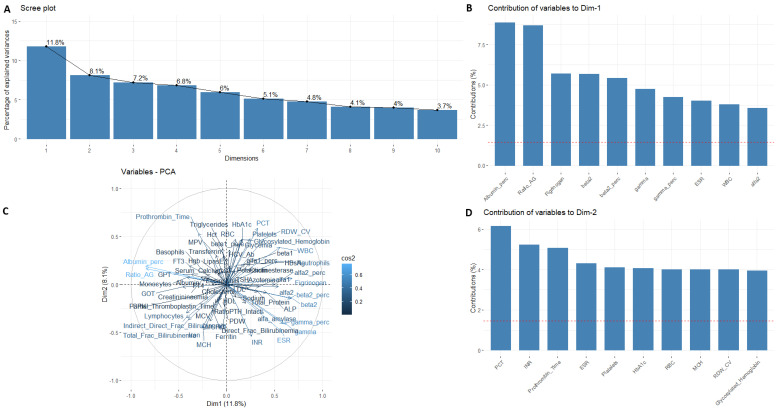
Scree plot of the PCA parameters. First, two components were selected (**A**); the contribution of variables of Component 1 (**B**), and Component 2 (**D**). Biplot of PCA on the data showing the projection of the data set on the PC1 × PC2 plane (**C**). Male patients.

**Figure 3:**
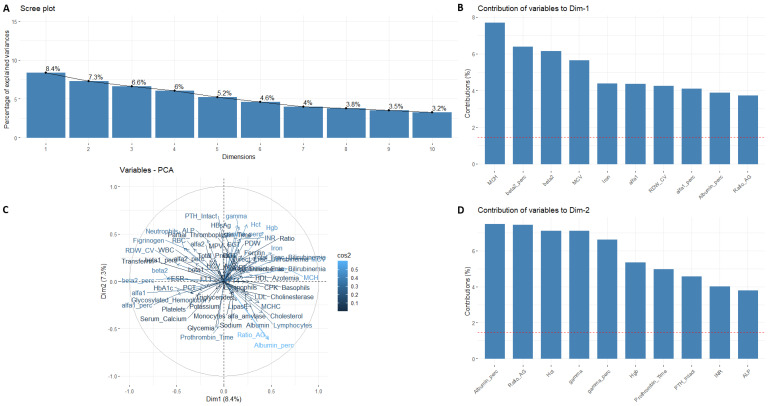
Scree plot of the PCA parameters. First, two components were selected (**A**); the contribution of variables of Component 1 (**B**), and Component 2 (**D**). Biplot of PCA on the data showing projection of the data set on the PC1 × PC2 plane (**C**). Female patients.

To summarize and visualize the information about prehospitalization blood parameters and create a profile of obese patients by gender and grade of steatosis, PCA was performed. Figure 2A shows a scree plot displaying the number of principal components. Firstly, two dimensions explain the 11.8% and 8.1% of variance for males and 8.4% and 7.3% for females, respectively (Figure 3A). These two dimensions were then visualized in a biplot (Figure 2C and Figure 3C) in which the variables coordinates are represented as projections inside the circle (i.e., Dimension 1 × Dimension 2 plane). A high value of variable contribution (called “cos2”) indicates a quality representation of the variable of the principal component and vice versa, a low value shows an imperfect representation. Variables close to the center of the plot were less important for the first components. This biplot shows a clear separation between parameters, with a high contribution of Albumin (%) and the A/G Ratio both for males and females (Figure 2B and Figure 3D). Figure 2C,D show the variable that constituted this component, for males and for females (Figure 3C,D).

**Figure 4:**
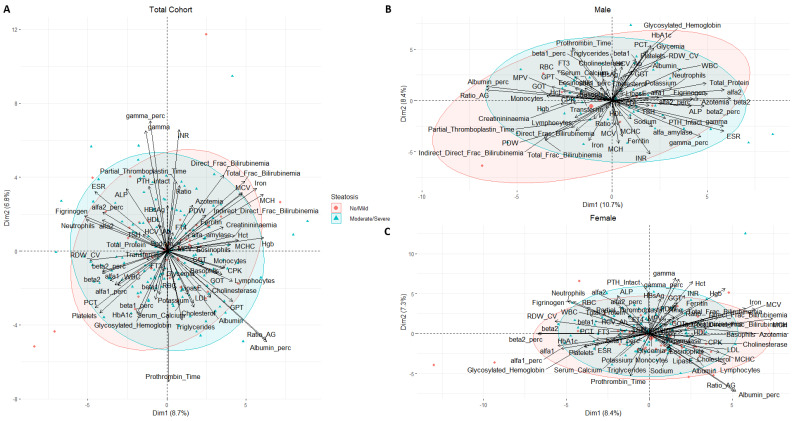
Biplot of PCA by steatosis in total (**A**), male (**B**), and female (**C**) cohort.

In the biplot (Figure 4), PCA analysis could not clearly identify a distinct steatosis-specific condition in the three groups analyzed (total, male and female cohorts). In the first two dimensions (Figure 4A), the variance explained was 8.7% and 6.8%, respectively, while it was 10.7% and 8.4% for the male and 8.4% and 7.3% for the female cohort.

## 4. Discussion

The aim of this study was to examine a new cohort of patients in southern Italy with pathological obesity and, in particular, to explore differences between genders in regard to their blood parameters.

The prevalence of overweight and obese men and women varies greatly within and between countries. Overall, more women than men were affected by obesity [18]. Differences due to men’s employment status and to their social context, as well as to the level of education and civil status, may work against the female sex. Added to these were bad habits, such as cigarette smoking [19]. Finally, these social differences were compounded by genetic, physiological, and hormonal profile differences in the patients analyzed. The differences between gender found in our cohort were reflected in the literature, although the possibility cannot be completely ruled out that obesity could amplify differences between the two genders.

With respect to blood parameters, plasma albumin concentrations did not differ between males and females [20] in healthy patients, but they were lower in our women with obesity, and this could be due to pathological obesity. Albumin levels were involved in appetite regulation because they could serve as carrier ligands for molecular substances, such as ghrelin, involved in stimulating appetite [21,22]. The same applies to PTH and PCT. Gender-related differences were not observed for WBC, neutrophils, and lymphocytes in the current study, unlike in previous findings [23], probably due to the pathological obesity condition. As demonstrated in literature, although its basis is still unclear, the A/G ratio was different by gender [24], but in this context, a lower ratio level (i.e., less albumin and more globulin) could be associated with a worse health condition in female patients. In the same health condition, higher beta globulin 2 was associated with peripheral arterial disease [25], and in our cohort, also with hypertension, that was a risk factor for vascular disorders [26]. ESR, instead, was associated with advancing age in females [27]. Glucose levels were lower in women, probably due to lower fasting plasma glucose in experiments conducted with the administration of glucose during the tolerance test [28]. We found lower triglyceride levels in female patients.

Estrogens could contribute to gender differences by directly influencing lipid metabolism through the suppression of gene expression and activity of lipoprotein lipase (LPL), the rate-limiting enzyme in triglyceride metabolism [29,30]; the modulation of lipolysis via an upregulation of α2-adrenergic receptors might contribute to lower TG concentrations [31] associated with the modulation of lipidic metabolism; females showed larger HDL particles than men, and this could justify the higher HDL levels [32].

Body composition differed between men and women. Men had more lean mass than women and were more likely to accumulate adipose tissue around the trunk and abdomen, whereas women usually accumulate adipose tissue around the hips and thighs [33]. According to these differences, women in this study had lower mean creatinine levels due to the lower muscle mass and meat intake [34] than men, who had a higher muscle energy metabolism than women of the same age [35,36]. Regarding serum levels of GGT, a higher level had been associated with a different distribution of fat in men compared to women [37,38]. Sex hormone fluctuations in females were responsible for lower iron and ferritin levels [39] that were also associated with lower concentrations of MCH, MCHC, PDW, and higher levels of transferrin. Platelets were also involved in these fluctuations, with increased platelets concentrations in females due to hormones or compensation due to menstruation [40,41].

Estradiol produced by the ovaries probably regulates the RBC levels, possibly affecting the nitric oxide (NO) synthesizing pathways [42].

Concerning lower FT3 levels in females, this finding could be due to lower testosterone levels than in males, which affect the peripheral (type 1) deiodinase as shown in a murine model [43,44], but it was also possible that testosterone played a role in the regulation of responses to thyroid hormone influenced by free dihydrotestosterone concentrations [45,46].

Genetic bases, on the other hand, likely justify the Hct levels, where the sequence-based polymorphism for erythropoietin could be partly responsible for this gender-based variation in Hct levels [47], while the immune response could justify the differences between genders regarding monocytes and eosinophils concentrations.

Obesity was well known to be responsible for a higher risk of steatosis [48], and we found by ultrasound examination that obesity was associated to steatosis and increased liver volume. We reported a sexually dimorphic disease and, interestingly, women had a reduced risk of steatosis compared to men of childbearing age, while after menopause women had a comparable prevalence of steatosis to men [49].

Our finding of the major prevalence of steatosis in males had been previously demonstrated [50]. Even if males had a higher prevalence of this disease in our cohort, worse clinical effects were demonstrated in females affected by obesity and steatosis. These results were apparently in contrast with those obtained in other studies performed in obese patients, where males had worse blood parameters [51], although the steatosis condition did not seem to influence the blood profile. Therefore, it seems that the effects of obesity per se were independent of those of steatosis and that the differences shown in this study were based on different genetic, hormone, or microbiota profiles. In fact, the axiom that “fatty people have fatty livers” was not correct because recent studies identified key molecular mediators involved in the progression of steatosis and obesity [52]. A balanced nutrition program diet was a good treatment in order to prepare the patient for surgery and a changed lifestyle.

Diet was important because it reduced liver volume, intraoperative complications, and operating time. Weight loss was also associated with reduction of respiratory failure and sleep apnea [53]. Furthermore, diet was the first step to keep psychological benefits and to try to understand if obese patients were intent to proceed to this new therapeutic path [54].

## 5. Conclusions

The present study shows gender differences in morbid obesity and related steatosis. Knowing the possible causes leading to these divergences in morbid obesity is important to try to reduce the impact of this discrimination on the psychological, social and metabolic health of obese patients. Understanding gender differences in obesity and steatosis status could have an important impact on the treatment of obesity and on planning community and individual interventions for the prevention and management of this condition. 

The average age of the patients was low, and the real effect on their health status was not yet evident. In the future, it could be interesting to evaluate a follow-up of this un-explored cohort to gain more information on patients undergoing bariatric surgery and evaluate how this procedure could be effective, firstly for losing weight and subsequently for improving their lifestyle and state of health. 

It would also be interesting to add the evaluation using instruments (DEXA or MRI) which evaluate the change in lean mass and fat mass in order to have a direct parameter on the efficacy not only of the dietary intervention but also of the surgical contribution.

The creation of personalized diets based on gender could increase efficacy because the dietary adherence was different by gender; women had greater sensitivity to the diet approach than men and ate a healthier diet. This different approach was reflected in the blood parameters, which improved after the diet.

Furthermore, the administration of psychological questionnaires could be useful to better understand the psychological status of patients eligible for bariatric surgery, compare the two genders and liver status, and to improve their emotional status.

## Figures and Tables

**Table 1:** Baseline epidemiological and clinical characteristics in obese patients undergoing bariatric surgery, by gender. Obesity Center cohort (*n* = 250).

Parameters *	Total Cohort (*n* = 250)	Males (*n* = 69)	Females (*n* = 181)	*p* ^^^
Age (y)	43.5 ± 11.9	42.4 ± 11.9	44.0 ± 11.9	0.45
Age Classes (%)				0.12 ^ψ^
Young Adult (≤25 y)	23 (9.2)	10 (14.5)	13 (7.2)	
Adult (26–44 y)	106 (42.4)	26 (37.7)	80 (44.2)	
Middle Aged (45–59 y)	99 (39.6)	30 (43.5)	69 (38.1)	
Elderly (≥60 y)	22 (8.8)	3 (4.3)	19 (10.5)	
Level of Education (%)				0.64 ^ψ^
None	5 (2.2)	1 (1.5)	4 (2.4)	
Elementary School	9 (3.9)	4 (6.0)	5 (3.1)	
Secondary school	173 (75.2)	51 (76.1)	122 (74.8)	
High school	41 (17.8)	10 (14.9)	31 (19.0)	
Degree	2 (0.9)	1 (1.5)	1 (0.6)	
Marital Status (%)				0.47 ^ψ^
Unmarried	44 (27.3)	13 (27.1)	31 (27.4)	
Married	103 (64.0)	33 (68.7)	70 (61.9)	
Separated	7 (4.3)	0 (0.0)	7 (6.2)	
Divorced	4 (2.5)	1 (2.1)	3 (2.6)	
Widow/er	3 (1.9)	1 (2.1)	2 (1.8)	
Smoking habit (%)				0.10 ^ψ^
No	121 (51.3)	31 (47.0)	90 (52.9)	
Ex	47 (19.9)	19 (28.8)	28 (16.5)	
Yes	68 (28.8)	16 (24.2)	52 (30.6)	
BMI (kg/m^2^)	46.6 ± 8.4	47.9 ± 10.2	46.1 ± 7.5	0.54
Alcohol Consumption (die) (%)				0.62 ^ψ^
None	148 (74.4)	43 (76.8)	105 (73.4)	
Occasional	51 (25.6)	13 (23.2)	38 (26.6)	
Physical Activity (%)				0.95 ^ψ^
Poor	161 (85.2)	47 (85.4)	114 (85.1)	
Occasional	28 (14.8)	8 (14.5)	20 (14.9)	
Employment (Yes) (%)	85 (59.7)	32 (82.0)	53 (51.5)	0.001
Diet (%)				0.30 ^ψ^
Ketogenic	164 (69.2)	49 (74.2)	115 (67.2)	
Low-Carb	56 (23.6)	15 (22.7)	41 (24.0)	
Hypocaloric	17 (7.2)	2 (3.0)	15 (8.8)	
Surgery (%)				0.39 ^ψ^
Sleeve	117 (47.8)	37 (55.2)	80 (44.9)	
Bypass	113 (46.1)	29 (43.3)	84 (47.2)	
LAGB	2 (0.8)	0 (0.0)	2 (1.1)	
SAGI	4 (1.6)	1 (1.5)	3 (1.7)	
Redo-Bypass	8 (3.3)	0 (0.0)	8 (4.5)	
Redo-Sleeve	1 (0.4)	0 (0.0)	1 (0.6)	
Hospitalization (days)	3.7 ± 2.7	3.7 ± 2.0	3.7 ± 3.0	0.65
*Blood tests*				
Total Protein (g/dL)	6.6 ± 0.6	6.6 ± 0.7	6.6 ± 0.6	0.85
Albumin (%)	53.3 ± 4.3	54.1 ± 4.6	53.0 ± 4.1	0.03
Alpha-1 (%)	2.7 ± 1.7	2.5 ± 0.4	2.9 ± 2.0	0.09
Alpha-2 (%)	12.2 ± 1.9	11.8 ± 1.9	12.4 ± 1.9	0.08
Beta-1 (%)	10.1 ± 2.0	9.9 ± 1.6	10.2 ± 2.2	0.27
Gamma (%)	14.7 ± 3.8	14.6 ± 3.4	14.8 ± 3.9	0.35
Ratio A/G	1.1 ± 0.2	1.2 ± 0.2	1.1 ± 0.2	0.03
Albumin (g/dL)	3.5 ± 0.4	3.6 ± 0.5	3.5 ± 0.4	0.19
Alpha-1 (g/dL)	0.2 ± 0.1	0.2 ± 0.0	0.2 ± 0.1	0.49
Alpha-2 (g/dL)	0.8 ± 0.1	0.8 ± 0.2	0.8 ± 0.1	0.20
Beta-1 (g/dL)	0.7 ± 0.2	0.7 ± 0.1	0.7 ± 0.2	0.52
Gamma (g/dL)	1.0 ± 0.3	1.0 ± 0.3	1.0 ± 0.3	0.33
Beta-2 (%)	6.9 ± 1.8	7.2 ± 1.4	6.7 ± 1.9	0.03
Beta-2 (g/dL)	0.5 ± 0.1	0.5 ± 0.1	0.4 ± 0.1	0.03
Glycemia (mg/dL)	96.4 ± 14.1	100.6 ± 16.1	94.7 ± 12.9	0.02
Triglycerides (mg/dL)	115.1 ± 49.7	127.2 ± 61.3	110.3 ± 43.5	0.04
Cholesterol (mg/mL)	182.8 ± 35.3	175.5 ± 28.7	185.7 ± 37.2	0.10
Total Fractional Bilirubinemia (mg/dL)	0.7 ± 0.3	0.7 ± 0.3	0.7 ± 0.3	0.10
Direct Fractional Bilirubinemia (mg/dL)	0.2 ± 0.1	0.2 ± 0.1	0.2 ± 0.1	0.93
Indirect Fractional Bilirubinemia (mg/dL)	0.5 ± 0.2	0.5 ± 0.2	0.5 ± 0.2	0.06
Azotemia (mg/dL)	35.1 ± 10.2	36.2 ± 9.3	34.7 ± 10.6	0.23
Creatininemia (mg/dL)	0.8 ± 0.1	0.9 ± 0.1	0.8 ± 0.1	<0.0001
CPK (U/L)	115.9 ± 69.3	146.0 ± 82.2	103.8 ± 59.5	0.0002
GOT (U/L)	23.2 ± 8.0	24.7 ± 8.7	22.6 ± 7.6	0.09
GGT (U/I)	22.7 ± 16.9	27.5 ± 19.5	20.7 ± 15.4	0.001
ALP (mU/mL)	157.7 ± 62.9	154.8 ± 60.5	158.9 ± 64.0	0.70
Iron (mg/dL)	59.3 ± 26.4	65.0 ± 23.7	57.0 ± 27.2	0.009
Ferritin (ng/mL)	82.4 ± 81.0	127.9 ± 77.3	64.3 ± 75.2	<0.0001
Transferrin (mg/dL)	319.3 ± 105.6	285.6 ± 55.3	332.8 ± 117.4	0.003
α—Amylase (UI/L)	54.8 ± 31.9	61.2 ± 53.0	52.3 ± 17.2	0.83
Sodium (mEq/L)	138.7 ± 2.4	138.5 ± 2.3	138.8 ± 2.5	0.35
Potassium (mEq/L)	4.1 ± 0.5	4.2 ± 0.4	4.1 ± 0.6	0.44
Serum Calcium (mg/dL)	8.9 ± 0.6	9.0 ± 0.7	8.9 ± 0.5	0.57
Cholinesterase (U/L)	10135.8 ± 2044.1	10632.0 ± 2290.6	9937.3 ± 1909.0	0.08
GPT (U/L)	32.1 ± 16.8	39.5 ± 20.1	29.2 ± 14.3	0.0003
Hepatitis C (IgG)	0.2 ± 1.1	0.1 ± 0.3	0.2 ± 1.3	0.30
HBsAg	0.1 ± 0.1	0.1 ± 0.0	0.1 ± 0.1	0.76
TSH (mUI/mL)	2.1 ± 1.7	1.6 ± 0.8	2.2 ± 1.9	0.008
FT3 (pg/mL)	3.3 ± 0.7	3.5 ± 0.5	3.3 ± 0.8	0.001
FT4 (ng/mL)	1.1 ± 0.2	1.1 ± 0.2	1.1 ± 0.2	0.14
Lipase (U/L)	36.5 ± 12.0	37.1 ± 12.7	36.2 ± 11.8	0.46
PTH Intact (pg/mL)	77.8 ± 36.6	70.2 ± 36.0	80.9 ± 36.5	0.03
LDL (mg/dL)	116.0 ± 32.4	113.3 ± 28.4	117.0 ± 33.9	0.70
HDL (mg/dL)	45.2 ± 11.8	39.0 ± 7.7	47.7 ± 12.2	<0.0001
Prothrombin Time—P (%)	108.3 ± 19.3	108.6 ± 16.1	108.2 ± 20.5	0.83
I.N.R.	1.0 ± 0.2	1.0 ± 0.1	1.0 ± 0.2	0.77
Partial Thromboplastin Time (sec)	27.8 ± 8.8	26.4 ± 5.0	28.4 ± 9.9	0.17
Ratio	1.0 ± 0.3	1.0 ± 0.2	1.0 ± 0.3	0.24
Fibrinogen-P (mg/dL)	383.2 ± 82.0	379.1 ± 109.4	384.9 ± 68.5	0.19
WBC (K/mcL)	7.2 ± 1.8	7.7 ± 2.1	7.0 ± 1.7	0.10
RBC (M/mcL)	4.9 ± 0.4	5.1 ± 0.4	4.8 ± 0.4	<0.0001
Hgb (g/dL)	13.7 ± 1.2	14.8 ± 0.9	13.3 ± 1.0	<0.0001
HCT (%)	41.6 ± 3.8	44.6 ± 3.2	40.5 ± 3.3	<0.0001
MCV (fL)	85.4 ± 5.8	86.7 ± 4.4	84.9 ± 6.2	0.13
MCH (pg)	28.2 ± 2.1	28.9 ± 1.4	27.9 ± 2.2	0.006
MCHC (g/dL)	33.0 ± 1.1	33.3 ± 1.1	32.8 ± 1.1	0.009
Platelets (K/mcL)	246.7 ± 55.9	233.3 ± 56.2	252.0 ± 55.1	0.03
RDW-CV (%)	14.3 ± 1.1	14.2 ± 1.1	14.4 ± 1.1	0.20
MPV (fL)	9.2 ± 1.0	9.1 ± 1.1	9.3 ± 0.9	0.35
PCT (%)	0.2 ± 0.0	0.2 ± 0.0	0.2 ± 0.0	0.009
PDW (%)	49.9 ± 6.2	51.1 ± 5.1	49.4 ± 6.5	0.02
Neutrophils (%)	62.8 ± 6.9	62.7 ± 6.6	62.8 ± 7.0	0.76
Lymphocytes (%)	27.3 ± 6.2	26.6 ± 5.8	27.5 ± 6.4	0.32
Monocytes (%)	5.6 ± 1.2	6.0 ± 1.2	5.4 ± 1.2	0.001
Eosinophils (%)	2.1 ± 1.2	2.3 ± 1.1	2.1 ± 1.2	0.03
Basophils (%)	0.5 ± 0.2	0.5 ± 0.3	0.5 ± 0.2	0.72
ESR	19.2 ± 12.9	12.1 ± 9.4	22.1 ± 12.9	<0.0001
HbA1c (%)	5.6 ± 0.7	5.7 ± 1.0	5.6 ± 0.6	0.18
Glycosylated Hemoglobin (mmol/mol)	38.5 ± 7.0	40.0 ± 7.5	37.9 ± 6.7	0.13
Blood Group (%)				0.25 ^ψ^
0	106 (44.5)	32 (48.5)	74 (43.0)	
A	95 (39.9)	28 (42.4)	67 (38.9)	
AB	11 (4.6)	3 (4.5)	8 (4.6)	
B	26 (10.9)	3 (4.5)	23 (13.4)	
Rh Factor (%)				0.63 ^ψ^
Negative	25 (10.4)	8 (11.9)	17 (9.8)	
Positive	215 (89.6)	59 (88.1)	156 (90.2)	
*Concomitant diseases*				
Allergy (Yes) (%)	89 (36.6)	18 (27.3)	71 (40.1)	0.06 ^ψ^
Hypertension (Yes) (%)	110 (44.0)	38 (55.1)	72 (39.8)	0.03 ^ψ^
Diabetes (Yes) (%)	97 (38.8)	33 (47.8)	64 (35.4)	0.07 ^ψ^
*Ultrasound Results*				
Liver Volume (%)				0.003 ^ψ^
Normal	89 (38.4)	14 (22.6)	75 (44.1)	
Increased	143 (61.6)	48 (77.4)	95 (55.9)	
Liver Margins (%)				0.23 ^ψ^
Smooth	197 (98.0)	45 (95.7)	152 (98.7)	
Irregular	4 (2.0)	2 (4.3)	2 (1.3)	
Steatosis (%)				0.006 ^ψ^
No/Mild	66 (28.4)	9 (14.7)	57 (33.3)	
Moderate/Severe	166 (71.5)	52 (85.2)	114 (66.7)	
Gallbladder Volume (%)				0.03 ^ψ^
Ablated	24 (10.7)	3 (5.2)	21 (12.6)	
Normal	183 (81.3)	47 (81.0)	136 (81.4)	
Expanse	13 (5.8)	4 (6.9)	9 (5.4)	
Contracted	1 (0.4)	1 (1.7)	0 (0.0)	
Scleroatrophic	4 (1.8)	3 (1.7)	1 (0.6)	
Gallbladder Stones (Yes) (%)	35 (17.2)	11 (19.0)	24 (16.4)	0.67 ^ψ^
Spleen Volume (%)				0.57 ^ψ^
Normal	214 (92.6)	56 (94.9)	158 (91.9)	
Increased	17 (7.4)	3 (5.1)	14 (8.1)	

* As mean and standard deviation (M ± SD) for continuous and percentage (%) for categorical variables. ^^^ Wilcoxon rank-sum test (Mann–Whitney), ^ψ^ chi-Square or Fisher’s test, where necessary. Abbreviations: BMI, body mass index; LAGB, adjustable gastric banding; SAGI, single anastomosis gastro-ileal bypass; ratio A/G, albumin/globulin; CPK, creatine kinase; GOT, aspartate amino transferase; GGT, gamma-glutamyl transferase; ALP, alkaline phosphatase; GPT, glutamic-pyruvic transaminase; HbsAg, hepatitis B surface antigen; TSH, thyroid stimulating hormone; FT3, triiodothyronine free; FT4, thyroxine; PTH intact, parathyroid hormone intact; LDL, low-density lipoprotein; HDL, high-density lipoprotein; I.N.R. international normalized ratio; WBC, white blood cells; RBC, red blood cell; Hgb, hemoglobin; HCT, hematocrit; MCV, mean corpuscular volume; MCH, mean corpuscular hemoglobin; MCHC, mean corpuscular hemoglobin concentration; RDW-CV, red cell distribution width—coefficient of variation; MPV, mean platelet volume; PCT, procalcitonin; PDW, platelet distribution width; ESR, red blood cells sedimentation rate; HbA1c, hemoglobin A1c.

**Table 2:** Baseline epidemiological and clinical characteristics of obese patients, by gender and steatosis (No/Yes) all undergoing to bariatric surgery. Obesity Center cohort (*n* = 250).

Parameters *	Total Cohort (*n* = 250)		*p* ^^^	Males (*n* = 69)		*p* ^^^	Females (*n* = 181)		*p* ^^^	*p* ^¥^	*p* ^†^
	No/Mild Steatosis (*n* = 66)	Moderate/ Severe Steatosis (*n* = 166)		No/Mild Steatosis (*n* = 9)	Moderate/ Severe Steatosis (*n* = 52)		No/Mild Steatosis (*n* = 57)	Moderate/ Severe Steatosis (*n* = 114)			
Age (y)	40.8 ± 12.0	44.6 ± 11.9	0.03	40.9 ± 16.3	42.7 ± 11.5	0.62	40.8 ± 11.3	45.5 ± 12.0	0.01	0.99	0.18
Age Classes (%)			0.50 ^ψ^			0.52 ^ψ^			0.32 ^ψ^	0.32 ^α^	0.22 ^β^
Young Adult (≤25 y)	7 (10.6)	15 (9.0)		2 (22.2)	7 (13.5)		5 (8.8)	8 (7.0)			
Adult (26–44 y)	32 (48.5)	65 (39.2)		3 (33.3)	20 (38.5)		29 (50.9)	45 (39.5)			
Middle Age (45–59 y)	23 (34.8)	70 (42.2)		3 (3.3)	23 (44.2)		20 (35.1)	47 (41.2)			
Old Age (≥ 60 y)	4 (6.1)	16 (9.6)		1 (11.1)	2 (3.8)		3 (5.3)	14 (12.3)			
Degree of Education (%)			0.65 ^ψ^			0.21 ^ψ^			0.71 ^ψ^	0.20 ^α^	0.66 ^β^
No	1 (1.7)	2 (1.3)		0 (0.0)	0 (0.0)		1 (2.0)	2 (1.9)			
Elementary School	3 (5.3)	5 (3.2)		2 (25.0)	2 (3.9)		1 (2.0)	3 (2.9)			
Secondary school	41 (71.9)	121 (77.6)		5 (62.5)	40 (78.4)		36 (73.5)	81 (77.1)			
High school	11 (19.3)	27 (17.3)		1 (12.5)	8 (15.7)		10 (20.4)	19 (18.1)			
Short Degree	1 (1.7)	1 (0.6)		0 (0.0)	1 (2.0)		1 (2.0)	0 (0.0)			
Marital Status (%)			<0.001 ^ψ^			0.15 ^ψ^			0.003 ^ψ^	0.47 ^α^	0.99 ^β^
Unmarried maiden	14 (34.1)	26 (24.5)		2 (40.0)	9 (25.7)		12 (33.3)	17 (23.9)			
Married	18 (43.9)	76 (71.7)		2 (40.0)	25 (71.4)		16 (44.4)	51 (71.8)			
Separate	6 (14.6)	1 (0.9)		0 (0.0)	0 (0.0)		6 (16.7)	1 (1.4)			
Divorced	1 (2.4)	3 (2.8)		0 (0.0)	1 (2.9)		1 (2.8)	2 (2.8)			
Widower	2 (4.9)	0 (0.0)		1 (20.0)	0 (0.0)		1 (2.8)	0 (0.0)			
Smoke (%)			0.47 ^ψ^			0.33 ^ψ^			0.53 ^ψ^	0.34 ^α^	0.10 ^β^
No	31 (51.7)	82 (51.6)		6 (75.0)	22 (43.1)		25 (48.1)	60 (55.6)			
Ex	9 (15.0)	34 (21.4)		1 (12.5)	16 (31.4)		8 (15.4)	18 (16.7)			
Yes	20 (33.3)	43 (27.0)		1 (12.5)	13 (25.5)		19 (36.5)	30 (27.8)			
BMI (Kg/m^2^)	44.1 ± 6.2	47.1 ± 8.4	0.02	46.4 ± 6.3	47.4 ± 9.9	0.93	43.7 ± 6.2	47.0 ± 7.7	0.008 ^ψ^	0.18	0.71
Alcohol Consumption (die) (%)			0.41 ^ψ^			0.66 ^ψ^			0.49 ^ψ^	0.99 ^α^	0.73 ^β^
No	41 (77.4)	5 (71.4)		6 (85.7)	33 (73.3)		35 (76.1)	62 (70.4)			
Sporadic	12 (22.6)	38 (28.6)		1 (14.3)	12 (26.7)		11 (23.9)	26 (29.5)			
Physical Activity (%)			0.16 ^ψ^			0.99 ^ψ^			0.10 ^ψ^	0.99 ^α^	0.51 ^β^
Poor	39 (78.0)	109 (86.5)		6 (85.7)	36 (83.7)		33 (76.7)	73 (87.9)			
Occasional	11 (22.0)	17 (13.5)		1 (14.3)	7 (16.3)		10 (23.3)	10 (15.0)			
Working Activity (Yes) (%)	25 (61.0)	57 (60.6)	0.97 ^ψ^	2 (66.7)	28 (84.8)	0.43 ^ψ^	23 (60.5)	29 (47.5)	0.21 ^ψ^	0.99 ^α^	<0.001 ^β^
Diet (%)			0.05 ^ψ^			0.99 ^ψ^			0.03 ^ψ^	0.39 ^α^	0.65 ^β^
Ketogenic	36 (59.0)	119 (75.3)		6 (85.7)	38 (74.5)		30 (55.6)	81 (75.7)			
Low-Carb	19 (31.1)	29 (18.3)		1 (14.3)	11 (21.6)		18 (33.3)	18 (16.8)			
Hypocaloric	6 (9.8)	10 (6.3)		0 (0.0)	2 (3.9)		6 (11.1)	8 (7.5)			
Surgery (%)			0.009 ^ψ^			0.72 ^ψ^			0.01 ^ψ^	0.72 ^α^	0.58 ^β^
Sleeve	21 (32.8)	87 (53.4)		4 (50.0)	29 (56.9)		17 (30.4)	58 (51.8)			
Bypass	38 (59.4)	68 (41.7)		4 (50.0)	22 (43.1)		34 (60.7)	46 (41.1)			
LAGB	2 (3.1)	0 (0.0)		0 (0.0)	0 (0.0)		2 (3.6)	0 (0.0)			
SAGI	0 (0.0)	2 (1.2)		0 (0.0)	0 (0.0)		0 (0.0)	2 (1.8)			
Redo-Bypass	3 (4.7)	5 (3.1)		0 (0.0)	0 (0.0)		3 (5.4)	5 (4.5)			
Redo-Sleeve	0 (0.0)	1 (0.6)		0 (0.0)	0 (0.0)		0 (0.0)	1 (0.9)			
Hospitalization (days)	3.3 ± 2.3	3.8 ± 2.9	0.001	3.1 ± 0.8	3.7 ± 2.2	0.60	3.3 ± 2.5	3.8 ± 3.2	0.002	0.55	0.62
*Blood tests*											
Total Protein (g/dL)	6.5 ± 0.7	6.7 ± 0.6	0.50	6.1 ± 1.2	6.7 ± 0.7	0.14	6.6 ± 0.7	6.7 ± 0.5	0.92	0.28	0.53
Albumin (%)	54.0 ± 4.1	53.1 ± 4.2	0.27	56.0 ± 3.5	54.2 ± 4.1	0.44	53.7 ± 4.1	52.6 ± 4.1	0.17	0.19	0.01
Alpha-1 (%)	3.3 ± 3.2	2.6 ± 0.8	0.45	2.5 ± 0.4	2.4 ± 0.3	0.90	3.4 ± 3.4	2.6 ± 0.9	0.67	0.46	0.10
Alpha-2 (%)	11.8 ± 1.6	12.3 ± 2.0	0.04	11.5 ± 2.2	11.8 ± 1.9	0.82	11.9 ± 1.6	12.6 ± 2.0	0.007	0.78	0.02
Beta-1 (%)	10.0 ± 2.1	10.2 ± 2.1	0.45	10.5 ± 3.3	9.9 ± 1.3	0.81	10.0 ± 2.0	10.3 ± 2.3	0.30	0.84	0.31
Gamma (%)	14.0 ± 4.4	14.9 ± 3.6	0.57	12.4 ± 1.8	14.6 ± 3.5	0.11	14.2 ± 4.6	15.1 ± 3.6	0.80	0.05	0.35
Ratio A/G	1.2 ± 0.2	1.1 ± 0.2	0.27	1.3 ± 0.2	1.2 ± 0.2	0.42	1.2 ± 0.2	1.1 ± 0.2	0.16	0.17	0.01
Albumin (g/dL)	3.5 ± 0.4	3.6 ± 0.4	0.60	3.3 ± 0.5	3.6 ± 0.4	0.15	3.5 ± 0.4	3.5 ± 0.4	0.73	0.42	0.04
Alpha-1 (g/dL)	0.2 ± 0.2	0.9 ± 0.1	0.72	0.1 ± 0.0	0.2 ± 0.0	0.02	0.2 ± 0.2	0.2 ± 0.1	0.68	0.03	0.97
Alpha-2 (g/dL)	0.8 ± 0.1	0.8 ± 0.1	0.03	0.7 ± 0.2	0.8 ± 0.1	0.50	0.8 ± 0.1	0.8 ± 0.1	0.01	0.72	0.10
Beta-1 (g/dL)	0.7 ± 0.2	0.7 ± 0.2	0.67	0.6 ± 0.2	0.7 ± 0.1	0.94	0.7 ± 0.2	0.7 ± 0.2	0.60	0.96	0.72
Gamma (g/dL)	0.9 ± 0.3	1.0 ± 0.3	0.28	0.8 ± 0.2	1.0 ± 0.3	0.09	0.9 ± 0.3	1.0 ± 0.2	0.43	0.09	0.29
Beta-2 (%)	6.8 ± 2.7	6.9 ± 1.3	0.22	6.9 ± 1.2	7.0 ± 1.2	0.72	6.8 ± 2.9	6.8 ± 1.3	0.41	0.55	0.16
Beta-2 (g/dL)	0.4 ± 0.2	0.5 ± 0.1	0.14	0.4 ± 0.1	0.5 ± 0.1	0.25	0.4 ± 0.2	0.4 ± 0.1	0.44	0.87	0.05
Glycemia (mg/dL)	93.7 ± 9.8	97.5 ± 15.3	0.14	92.4 ± 5.5	102.8 ± 16.9	0.09	93.9 ± 10.3	0.9 ± 13.8	0.71	0.90	0.007
Triglycerides (mg/dL)	94.4 ± 28.1	122.2 ± 54.0	0.0005	91.1 ± 25.2	132.7 ± 66.0	0.08	95.0 ± 28.8	117.4 ± 47.0	0.006	0.92	0.14
Cholesterol (mg/mL)	185.1 ± 39.9	182.2 ± 34.5	0.88	184.3 ± 34.6	174.4 ± 28.0	0.29	185.2 ± 41.0	185.8 ± 36.7	0.62	0.87	0.08
Total Fractional Bilirubinemia (mg/dL)	0.7 ± 0.3	0.7 ± 0.3	0.08	0.9 ± 0.4	0.7 ± 0.3	0.04	0.7 ± 0.3	0.6 ± 0.3	0.21	0.07	0.30
Direct Fractional Bilirubinemia (mg/dL)	0.2 ± 0.1	0.2 ± 0.1	0.26	0.2 ± 0.1	0.2 ± 0.2	0.35	0.2 ± 0.1	0.2 ± 0.2	0.44	0.75	0.60
Indirect Fractional Bilirubinemia (mg/dL)	0.5 ± 0.3	0.5 ± 0.2	0.13	0.7 ± 0.4	0.5 ± 0.2	0.03	0.5 ± 0.2	0.5 ± 0.2	0.26	0.04	0.21
Azotemia (mg/dL)	36.1 ± 12.5	34.8 ± 9.4	0.81	34.0 ± 8.6	36.8 ± 9.3	0.39	36.5 ± 13.0	33.9 ± 9.4	0.40	0.71	0.07
Creatininaemia (mg/dL)	0.8 ± 0.1	0.8 ± 0.1	0.40	0.9 ± 0.1	0.9 ± 0.1	0.73	0.8 ± 0.1	0.8 ± 0.1	0.10	0.008	<0.0001
CPK (U/L)	101.0 ± 50.7	122.3 ± 73.7	0.15	122.7 ± 46.2	152.6 ± 84.4	0.45	97.4 ± 51.1	108.2 ± 63.8	0.51	0.09	0.001
GOT (U/L)	20.9 ± 6.2	23.9 ± 8.2	0.02	19.9 ± 4.9	25.4 ± 8.9	0.11	21.0 ± 6.4	23.2 ± 7.9	0.12	0.82	0.18
GGT (U/I)	18.2 ± 13.6	23.6 ± 17.8	0.01	19.9 ± 10.8	28.1 ± 20.5	0.27	17.9 ± 14.1	21.5 ± 16.1	0.09	0.44	0.007
ALP (mU/mL)	151.3 ± 69.7	160.6 ± 61.6	0.29	134.1 ± 77.2	155.1 ± 60.9	0.53	154.1 ± 68.9	163.1 ± 62.1	0.29	0.61	0.42
Iron (mg/dL)	59.3 ± 22.8	59.4 ± 28.2	0.85	75.7 ± 19.3	64.3 ± 24.6	0.09	56.7 ± 22.4	57.1 ± 29.6	0.82	0.03	0.04
Ferritin (ng/mL)	59.4 ± 72.4	86.4 ± 80.1	0.001	177.9 ± 114.7	112.8 ± 61.2	0.18	40.1 ± 38.9	74.1 ± 85.1	0.001	0.0002	<0.0001
Transferrin (mg/dL)	348.0 ± 162.6	312.5 ± 80.6	0.14	280.4 ± 61.6	286.5 ± 56.7	0.99	359.0 ± 171.5	324.6 ± 87.2	0.29	0.09	0.02
α—Amylase (UI/L)	50.0 ± 17.1	53.2 ± 19.6	0.44	53.7 ± 12.9	54.3 ± 24.9	0.32	49.4 ± 17.7	52.7 ± 16.7	0.19	0.35	0.38
Sodium (mEq/L)	139.0 ± 2.5	138.7 ± 2.4	0.63	138.7 ± 1.6	138.5 ± 2.5	0.63	139.0 ± 2.6	138.8 ± 2.4	0.87	0.94	0.35
Potassium (mEq/L)	4.3 ± 0.8	4.1 ± 0.4	0.41	4.5 ± 0.5	4.1 ± 0.4	0.10	4.2 ± 0.8	4.1 ± 0.4	0.80	0.04	0.83
Serum Calcium (mg/dL)	8.9 ± 0.5	8.9 ± 0.6	0.66	9.2 ± 0.6	9.0 ± 0.8	0.35	8.9 ± 0.5	8.9 ± 0.5	0.79	0.24	0.57
Cholinesterase (U/L)	9425.4 ± 2117.7	10,368.4 ± 2022.8	0.01	9557.1 ± 2671.4	10,763.0 ± 2308.5	0.31	9403.9 ± 2051.7	10,184.7 ± 1859.1	0.04	0.97	0.26
GPT (U/L)	25.9 ± 14.4	33.4 ± 16.1	0.0004	25.3 ± 6.9	40.2 ± 19.6	0.04	26.0 ± 15.3	30.2 ± 13.2	0.01	0.45	0.002
Hepatitis C (IgG)	0.3 ± 1.5	0.2 ± 0.9	0.43	0.0 ± 0.0	0.1 ± 0.3	0.10	0.3 ± 1.7	0.2 ± 1.1	0.77	0.08	0.61
HBsAg	0.1 ± 0.0	0.1 ± 0.1	0.96	0.1 ± 0.2	0.1 ± 0.0	0.22	0.1 ± 0.0	0.1 ± 0.1	0.56	0.19	0.76
TSH (mUI/mL)	2.4 ± 2.5	2.0 ± 1.4	0.12	1.9 ± 1.0	1.6 ± 0.8	0.62	2.5 ± 2.6	2.1 ± 1.6	0.30	0.35	0.008
FT3 (pg/mL)	3.4 ± 0.9	3.4 ± 0.7	0.99	3.4 ± 0.5	3.5 ± 0.5	0.47	3.4 ± 1.0	3.3 ± 0.7	0.39	0.59	0.001
FT4 (ng/mL)	1.2 ± 0.3	1.1 ± 0.2	0.48	1.2 ± 0.2	1.1 ± 0.2	0.49	1.2 ± 0.3	1.1 ± 0.2	0.78	0.79	0.14
Lipase (U/L)	35.9 ± 14.3	36.9 ± 11.5	0.26	33.9 ± 4.7	38.8 ± 13.5	0.33	36.2 ± 15.3	36.1 ± 10.3	0.61	0.82	0.46
PTH Intact (pg/mL)	69.9 ± 33.5	82.4 ± 37.3	0.02	56.3 ± 25.5	74.2 ± 37.1	0.27	72.1 ± 34.3	86.2 ± 37.0	0.01	0.11	0.03
LDL (mg/dL)	114.4 ± 37.7	116.1 ± 31.1	0.52	109.0 ± 46.8	111.9 ± 24.3	0.75	115.2 ± 36.6	118.1 ± 33.8	0.36	0.73	0.70
HDL (mg/dL)	48.0 ± 10.2	44.2 ± 11.7	0.02	42.7 ± 6.9	38.9 ± 7.9	0.21	48.8 ± 10.5	46.7 ± 12.3	0.22	0.14	<0.0001
Prothrombin Time—P (%)	105.8 ± 18.5	108.4 ± 19.4	0.41	111.2 ± 13.8	108.4 ± 17.2	0.70	105.0 ± 19.1	108.4 ± 20.5	0.30	0.72	0.83
I.N.R.	1.0 ± 0.4	1.0 ± 0.1	0.42	0.9 ± 0.0	1.0 ± 0.1	0.66	1.0 ± 0.4	1.0 ± 0.1	0.29	0.69	0.77
Partial Thromboplastin Time (sec)	27.9 ± 5.5	28.2 ± 10.0	0.26	25.2 ± 2.6	27.1 ± 5.3	0.35	28.3 ± 5.7	28.7 ± 11.5	0.17	0.06	0.17
Ratio	1.0 ± 0.2	1.0 ± 0.3	0.20	0.9 ± 0.1	0.1 ± 0.2	0.55	1.0 ± 0.2	1.0 ± 0.4	0.13	0.13	0.24
Fibrinogen-P (mg/dL)	371.5 ± 65.8	383.9 ± 79.2	0.32	367.2 ± 64.1	367.3 ± 95.3	0.79	372.2 ± 66.7	391.6 ± 69.7	0.10	0.97	0.19
WBC (K/mcL)	6.6 ± 1.9	7.4 ± 1.7	0.01	8.3 ± 2.7	7.4 ± 1.8	0.56	6.3 ± 1.7	7.3 ± 1.7	0.004	0.08	0.10
RBC (M/mcL)	4.8 ± 0.4	4.9 ± 0.4	0.02	5.0 ± 0.3	5.1 ± 0.3	0.60	4.7 ± 0.4	4.8 ± 0.4	0.19	0.04	<0.0001
Hgb (g/dL)	13.4 ± 1.0	13.8 ± 1.2	0.05	14.9 ± 0.8	14.8 ± 1.0	0.82	13.2 ± 0.9	13.4 ± 1.1	0.48	0.0004	<0.0001
HCT (%)	40.8 ± 3.43	41.9 ± 3.8	0.03	45.1 ± 4.0	44.3 ± 3.1	0.76	40.1 ± 2.8	40.7 ± 3.5	0.23	0.002	<0.0001
MCV (fL)	85.4 ± 5.7	85.5 ± 6.0	0.92	89.2 ± 4.6	86.7 ± 4.4	0.20	84.8 ± 5.6	95.0 ± 6.6	0.79	0.05	0.13
MCH (pg)	28.1 ± 2.1	28.2 ± 2.1	0.90	29.4 ± 1.3	28.9 ± 1.4	0.43	27.9 ± 2.2	27.8 ± 2.3	0.84	0.07	0.006
MCHC (g/dL)	32.9 ± 1.1	32.9 ± 1.1	0.98	33.0 ± 1.3	33.4 ± 1.1	0.79	32.9 ± 1.1	32.7 ± 1.1	0.50	0.38	0.009
Platelets (K/mcL)	249.6 ± 58.6	246.5 ± 56.4	0.99	250.7 ± 68.1	229.7 ± 57.2	0.54	249.4 ± 57.8	254.3 ± 54.6	0.51	0.89	0.03
RDW-CV (%)	14.3 ± 1.1	14.3 ± 1.1	0.76	14.6 ± 1.6	14.0 ± 1.0	0.37	14.3 ± 1.0	14.4 ± 1.2	0.74	0.94	0.20
MPV (fL)	9.2 ± 1.0	9.2 ± 1.0	0.65	9.6 ± 1.1	9.0 ± 1.1	0.37	9.1 ± 1.0	9.3 ± 0.9	0.29	0.35	0.35
PCT (%)	0.2 ± 0.0	0.2 ± 0.0	0.83	0.2 ± 0.1	0.2 ± 0.0	0.15	0.2 ± 0.0	0.2 ± 0.0	0.39	0.73	0.009
PDW (%)	47.8 ± 5.4	50.5 ± 6.2	0.01	50.3 ± 4.4	51.5 ± 5.1	0.51	47.4 ± 5.5	50.1 ± 6.7	0.04	0.12	0.002
Neutrophils (%)	61.6 ± 6.9	63.0 ± 6.9	0.13	61.5 ± 4.3	62.0 ± 6.5	0.87	61.6 ± 7.2	63.4 ± 7.0	0.09	0.91	0.76
Lymphocytes (%)	28.5 ± 5.9	27.0 ± 6.3	0.05	28.1 ± 4.1	27.0 ± 5.9	0.49	28.6 ± 6.2	27.0 ± 6.6	0.08	0.61	0.32
Monocytes (%)	5.6 ± 1.1	5.6 ± 1.2	0.59	6.1 ± 0.5	6.0 ± 1.3	0.74	5.6 ± 1.2	5.4 ± 1.1	0.32	0.17	0.001
Eosinophils (%)	2.1 ± 1.2	2.2 ± 1.2	0.76	2.1 ± 0.6	2.4 ± 1.1	0.66	2.1 ± 1.3	2.0 ± 1.2	0.76	0.62	0.03
Basophils (%)	0.5 ± 0.2	0.5 ± 0.2	0.39	0.5 ± 0.3	0.5 ± 0.3	0.99	0.5 ± 0.2	0.5 ± 0.2	0.31	0.83	0.72
ESR	20.0 ± 13.6	19.0 ± 12.6	0.63	8.5 ± 7.3	11.8 ± 8.7	0.25	21.7 ± 13.5	22.3 ± 12.8	0.69	0.01	<0.0001
HbA1c (%)	5.5 ± 0.7	5.7 ± 0.8	0.04	5.3 ± 0.4	5.8 ± 1.1	0.01	5.6 ± 0.7	5.6 ± 0.6	0.38	0.14	0.18
Glycosylated Hemoglobin (mmol/mol)	37.0 ± 7.3	39.0 ± 6.9	0.02	34.1 ± 4.8	41.2 ± 7.4	0.009	37.5 ± 7.7	38.1 ± 6.4	0.31	0.15	0.13
Blood Group (%)			0.15 ^ψ^			0.64 ^ψ^			0.04 ^ψ^	0.71 ^α^	0.25 ^β^
0	24 (37.5)	72 (45.9)		4 (44.4)	24 (49.0)		20 (36.4)	48 (44.4)			
A	34 (53.1)	58 (36.9)		4 (44.4)	22 (44.9)		30 (54.5)	36 (33.3)			
AB	2 (3.1)	7 (4.5)		0 (0.0)	1 (2.0)		2 (3.6)	6 (5.6)			
B	4 (6.2)	20 (12.7)		1 (11.1)	2 (4.1)		3 (5.4)	18 (16.7)			
Rh Factor (%)			0.88 ^ψ^			0.99 ^ψ^			0.84 ^ψ^	0.99 ^α^	0.63 ^β^
Negative	6 (9.4)	16 (10.1)		1 (11.1)	5 (10.0)		5 (9.1)	11 (10.1)			
Positive	58 (9.6)	143 (89.9)		8 (88.9)	45 (90.0)		50 (90.9)	98 (89.9)			
*Pathologies*											
Allergy (Yes) (%)	21 (33.3)	60 (37.0)	0.60 ^ψ^	2 (25.0)	11 (22.0)	0.99 ^ψ^	19 (34.5)	49 (43.7)	0.25 ^ψ^	0.71 ^α^	0.06 ^β^
Hypertension (Yes) (%)	29 (43.9)	73 (44.0)	0.99 ^ψ^	5 (55.5)	29 (55.8)	0.99 ^ψ^	24 (42.1)	44 (38.6)	0.66 ^ψ^	0.49 ^α^	0.03 ^β^
Diabetes (Yes) (%)	26 (39.4)	61 (36.7)	0.71 ^ψ^	4 (44.4)	26 (50.0)	0.99 ^ψ^	22 (38.6)	35 (30.7)	0.31 ^ψ^	0.73 ^α^	0.07 ^β^
*Ultrasound Results*											
Liver Volume (%)			<0.001 ^ψ^			<0.001 ^ψ^			<0.001 ^ψ^	0.09 ^α^	0.003 ^β^
Normal	61 (93.8)	27 (16.5)		7 (77.8)	6 (11.8)		54 (96.4)	21 (18.6)			
Increased	4 (6.1)	137 (83.5)		2 (22.2)	45 (88.2)		2 (3.6)	92 (81.4)			
Liver Margins (%)			0.99 ^ψ^			0.35 ^ψ^			0.55 ^ψ^	0.14 ^α^	0.23 ^β^
Smooth	61 (98.4)	136 (97.8)		8 (88.9)	37 (97.4)		53 (100.0)	99 (98.0)			
Irregular	1 (1.6)	3 (2.2)		1 (11.1)	1 (2.6)		0 (0.0)	2 (2.0)			
Gallbladder Volume (%)			0.98 ^ψ^			0.82 ^ψ^			0.63 ^ψ^	0.39 ^α^	0.03 ^β^
Ablated	7 (11.3)	16 (10.1)		0 (0.0)	2 (4.3)		7 (13.2)	14 (12.5)			
Normal	51 (82.3)	132 (83.0)		8 (88.8)	39 (83.0)		43 (81.1)	93 (83.0)			
Expanse	3 (4.8)	7 (4.4)		1 (11.1)	2 (4.3)		2 (3.8)	5 (4.5)			
Contracted	0 (0.0)	1 (0.6)		0 (0.0)	1 (2.1)		0 (0.0)	0 (0.0)			
Scleroatrophic	1 (1.6)	3 (1.9)		0 (0.0)	3 (6.4)		1 (1.9)	0 (0.0)			
Gallbladder Stones (Yes) (%)	5 (8.9)	30 (20.7)	0.05 ^ψ^	0 (0.0)	11 (23.4)	0.18 ^ψ^	5 (10.6)	19 (19.4)	0.18 ^ψ^	0.58 ^α^	0.67 ^β^
Spleen Volume (%)			0.55 ^ψ^			0.99 ^ψ^			0.44 ^ψ^	0.58 ^α^	0.57 ^β^
Normal	60 (90.9)	151 (93.2)		9 (100.0)	46 (93.9)		51 (89.5)	105 (92.9)			
Increased	6 (9.1)	11 (6.8)		0 (0.0)	3 (6.1)		6 (10.5)	8 (7.1)			

* As mean and standard deviation (M ± SD) for continuous variables, and percentage (%) for categorical. ^^^ Wilcoxon rank-sum test (Mann–Whitney), ^¥^ Wilcoxon rank-sum test (Mann–Whitney) between males and females without steatosis, ^†^ Wilcoxon rank-sum test (Mann–Whitney) between males and females with steatosis, ^ψ^ chi-square or Fisher’s test, where necessary, ^α^ chi-square or Fisher’s test, where necessary between males and females without steatosis, ^β^ chi-square or Fisher’s test, where necessary between males and females with steatosis. Abbreviations: BMI, body mass index; LAGB, adjustable gastric banding; SAGI, single anastomosis gastro-ileal bypass; ratio A/G, albumin/globulin; CPK, creatine kinase; GOT, aspartate aminotransferase; GGT, gamma-glutamyl transferase; ALP, alkaline phosphatase; GPT, glutamic-pyruvic transaminase; HbsAg, hepatitis B surface antigen; TSH, thyroid stimulating hormone; FT3, triiodothyronine free; FT4, thyroxine; PTH intact, parathyroid hormone intact; LDL, low-density lipoprotein; HDL, high-density lipoprotein; I.N.R. international normalized ratio; WBC, white blood cells; RBC, red blood cell; Hgb, hemoglobin; HCT, hematocrit; MCV, mean corpuscular volume; MCH, mean corpuscular hemoglobin; MCHC, mean corpuscular hemoglobin concentration; RDW-CV, red cell distribution width—coefficient of variation; MPV, mean platelet volume; PCT, procalcitonin; PDW, platelet distribution width; ESR, red blood cells sedimentation rate; HbA1c, hemoglobin A1c.

**Table 3:** Comparison of BMI and blood parameters by gender, between prehospitalization and surgery in obese patients receiving diet to lose weight before bariatric surgery.

Parameters *	Males		*p* ^^^	Females		*p* ^^^
	Prehospitalization	Surgery		Prehospitalization	Surgery	
BMI (Kg/m^2^)	47.9 ± 10.2	49.1 ± 10.2	0.17	46.1 ± 7.5	45.1 ± 7.3	0.09
Glycemia (mg/dL)	100.6 ± 16.1	99.6 ± 23.2	0.79	94.7 ± 12.9	97.3 ± 18.9	0.11
Fractional Total Bilirubinemia (mg/dL)	0.7 ± 0.3	0.8 ± 0.3	0.09	0.7 ± 0.3	0.7 ± 0.3	0.46
Direct fractional bilirubinemia (mg/dL)	0.2 ± 0.1	0.2 ± 0.1	0.19	0.2 ± 0.1	0.2 ± 0.1	0.86
Indirect fractional bilirubinemia (mg/dL)	0.5 ± 0.2	0.6 ± 0.3	0.18	0.5 ± 0.2	0.5 ± 0.3	0.74
Azotemia (mg/dL)	36.2 ± 9.3	31.8 ± 15.2	0.0002	34.7 ± 10.6	28.0 ± 11.4	<0.0001
Creatininaemia (mg/dL)	0.9 ± 0.1	0.9 ± 0.2	0.009	0.8 ± 0.1	0.7 ± 0.2	<0.0001
GOT (U/L)	24.7 ± 8.7	25.6 ± 9.3	0.79	22.6 ± 7.6	27.8 ± 18.1	<0.0001
GGT (U/I)	27.5 ± 19.5	23.8 ± 18.4	0.002	20.3 ± 15.4	18.9 ± 19.8	<0.0001
ALP (mU/mL)	154.8 ± 60.5	115.3 ± 55.8	<0.0001	158.9 ± 64.0	115.5 ± 52.3	<0.0001
Sodium (mEq/L)	138.5 ± 2.3	140.4 ± 3.5	0.02	138.8 ± 2.5	140.2 ± 4.2	0.001
Potassium (mEq/L)	4.2 ± 0.4	4.2 ± 0.4	0.08	4.1 ± 0.6	4.2 ± 0.5	0.99
GPT (U/L)	39.5 ± 20.1	39.1 ± 22.6	0.35	29.2 ± 14.3	34.7 ± 29.1	0.23
Lipase (U/L)	37.1 ± 12.7	30.9 ± 14.2	0.001	36.2 ± 11.8	30.0 ± 13.9	<0.0001
Prothrombin Time—P (%)	108.6 ± 16.1	103.0 ± 11.6	0.34	108.2 ± 20.5	94.8 ± 18.4	0.0005
I.N.R.	1.0 ± 0.1	1.0 ± 0.1	0.34	1.0 ± 0.2	1.0 ± 0.1	0.0003
Partial Thromboplastin Time (sec)	26.4 ± 5.0	25.7 ± 3.4	0.18	28.4 ± 9.9	28.5 ± 13.2	0.99
Ratio	1.0 ± 0.2	0.9 ± 0.1	0.99	1.0 ± 0.3	1.1 ± 0.5	0.14
WBC (K/mcL)	7.7 ± 2.1	10.6 ± 2.8	<0.0001	7.0 ± 1.7	10.0 ± 2.8	<0.0001
RBC (M/mcL)	5.1 ± 0.4	4.8 ± 0.5	<0.0001	4.8 ± 0.4	4.4 ± 0.4	<0.0001
Hgb (g/dL)	14.8 ± 0.9	13.8 ± 1.3	<0.0001	13.3 ± 1.0	12.4 ± 1.1	<0.0001
HCT (%)	44.6 ± 3.2	41.6 ± 4.6	<0.0001	40.5 ± 3.3	37.6 ± 3.5	<0.0001
MCV (fL)	86.7 ± 4.4	86.9 ± 6.4	0.05	84.9 ± 6.2	84.9 ± 6.5	0.03
MCH (pg)	28.9 ± 1.4	28.8 ± 1.8	0.36	27.9 ± 2.2	28.0 ± 2.3	0.80
MCHC (g/dL)	33.3 ± 1.1	33.2 ± 1.7	0.24	32.8 ± 1.1	33.0 ± 1.2	0.09
Platelets (K/mcL)	233.3 ± 56.2	210.8 ± 57.1	<0.0001	252.0 ± 55.1	229.2 ± 53.9	<0.0001
RDW-CV (%)	14.2 ± 1.1	13.9 ± 1.1	0.42	14.4 ± 1.1	14.2 ± 1.2	0.03
MPV (fL)	9.1 ± 1.1	9.4 ± 1.0	0.14	9.3 ± 0.9	9.3 ± 1.0	0.99
PCT (%)	0.2 ± 0.1	0.2 ± 0.1	0.002	0.2 ± 0.1	0.2 ± 0.1	<0.0001
PDW (%)	51.1 ± 5.1	51.2 ± 6.3	0.09	49.4 ± 6.5	49.3 ± 6.3	0.19
Neutrophils (%)	62.7 ± 6.6	78.8 ± 6.9	<0.0001	62.8 ± 7.0	79.2 ± 7.2	<0.0001
Lymphocytes (%)	26.6 ± 5.8	12.8 ± 5.0	<0.0001	27.5 ± 6.4	13.6 ± 5.9	<0.0001
Monocytes (%)	6.0 ± 1.2	6.2 ± 1.7	0.09	5.4 ± 1.2	5.4 ± 1.5	0.62
Eosinophils (%)	2.3 ± 1.1	0.7 ± 0.7	<0.0001	2.1 ± 1.2	0.7 ± 0.6	<0.0001
Basophils (%)	0.5 ± 0.3	0.2 ± 0.1	<0.0001	0.5 ± 0.2	0.2 ± 0.2	<0.0001

* As mean and standard Deviation (M ± SD) for continuous variables. ^^^ Wilcoxon matched-pairs signed-rank test. Abbreviations: BMI, body mass index; GOT, aspartate aminotransferase; GGT, gamma-glutamyl transferase; ALP, alkaline phosphatase; GPT, glutamic-pyruvic transaminase; HbsAg, hepatitis B surface antigen; TSH, thyroid stimulating hormone; FT3, triiodothyronine free; FT4, thyroxine; PTH intact, parathyroid hormone intact; LDL, low-density lipoprotein; HDL, high-density lipoprotein; I.N.R. international normalized ratio; WBC, white blood cells; RBC, red blood cell; Hgb, hemoglobin; HCT, hematocrit; MCV, mean corpuscular volume; MCH, mean corpuscular hemoglobin; MCHC, mean corpuscular hemoglobin concentration; RDW-CV, red cell distribution width—coefficient of variation; MPV, mean platelet volume; PCT, procalcitonin; PDW, platelet distribution width; ESR, red blood cells sedimentation rate; HbA1c, hemoglobin A1c.

## Data Availability

Not applicable.

## Supplementary Materials

The following supporting information can be downloaded at: https://www.mdpi.com/article/10.3390/nu15102381/s1, Table S1: Daily intake nutrients in different dietary regimen administered to the patient after to bariatric surgery.

## References

[1] Mayoral L.P., Andrade G.M., Mayoral E.P., Huerta T.H., Canseco S.P., Rodal Canales F.J., Cabrera-Fuentes H.A., Cruz M.M., Pérez Santiago A.D., Alpuche J.J. (2020). Obesity subtypes, related biomarkers & heterogeneity. Indian J. Med. Res..

[2] D’Errico M., Pavlova M., Spandonaro F. (2022). The economic burden of obesity in Italy: A cost-of-illness study. Eur. J. Health Econ..

[3] Piché M.E., Tchernof A., Despés J.P. (2020). Obesity phenotypes, diabetes, and cardiovascular diseases. Circ Res..

[4] Apovian C.M. (2016). Obesity: Definition, comorbidities, causes, and burden. Am. J. Manag. Care..

[5] Foschi D., Moroni R., De Luca M., Sarro G., Bernante P., Zappa M.A., Moroni R., Navarra G., Foletto M., Ceriani V. (2016). Linee guida di chirurgia dell’obesità. Società Italiana di Chirurgia Dell’Obesità e Delle Malattie (SICOB).

[6] D’Angela D., Cambiano C., Glorioso V. (2020). PariSanità-Osservatorio per l’equità di accesso alle prestazioni. Assobiomedica C.R.E.A. Sanità.

[7] Breznikar B., Dinevski D. (2009). Bariatric surgery for morbid obesity: Pre-operative assessment, surgical techniques, and post-operative monitoring. J. Int. Med. Res..

[8] Buchwald H., Oien D.M. (2009). Metabolic/bariatric surgery worldwide 2008. Obes. Surg..

[9] Reoch J., Mottillo S., Shimony A., Filion K.B., Christou N.V., Joseph L., Poirier P., Eisenberg M.J. (2011). Safety of laparoscopic vs. open bariatric surgery: A systematic review and meta-analysis. Arch. Surg..

[10] Kappor N., Arora S., Kalra S. (2021). Gender disparities in people living with obesity—An unchartered territory. J. Midlife Health..

[11] Shekelle P.G., Newberry S., Maglione M., Maglione M., Li Z., Yermilov I., Hilton L., Suttorp M., Maggard M., Carter J. (2008). Bariatric surgery in women of reproductive age: Special concerns for pregnancy. Evid. Rep. Technol. Assess..

[12] Dal Prà C., Fabris R. (2020). Obesity and gender differences. Ital. J. Gender-Specific Med..

[13] Wang D.Q.H., Portincasa P., Brend A., Neuschwander-Tetri B.A. (2013). Steatosis in the liver. Compr. Physiol..

[14] Denzer C., Thiere D., Muche R., Koening W., Mayer H., Kratzer W., Wabitsch M. (2009). Gender-specific prevalences of fatty liver in obese children and adolescents: Roles of body fat distribution, sex steroids, and insulin resistance. J. Clin. Endocrinol. Metab..

[15] Younossi Z.M., Stepanova M., Negro F., Hallaji S., Younossi Y., Lam B., Srishord M. (2012). Nonalcoholic fatty liver disease in lean individuals in the United States. Medicine.

[16] SICOB Indagine Conoscitiva Sulla Riduzione Dell’attività di Chirurgia Bariatrica COVID-19 Correlata. https://www.sicob.org/09_covid/chirurgia.html.

[17] Qayyum A., Chen D.M., Breiman R.S., Westphalen A.C., Yeh B.M., Jones K.D., Lu Y., Coakley F.V., Callen P.W. (2009). Evaluation of diffuse liver steatosis by ultrasound, computed tomography, and magnetic resonance imaging: Which modality is best?. Clin. Imaging.

[18] Kanter R., Caballero B. (2012). Global gender disparities in obesity: A review. Adv. Nutr..

[19] Pepino M.Y., Mennella J.A. (2014). Cigarette smoking and obesity are associated with decreased fat perception in women. Comp. Study.

[20] Schwab C., Paar M., Fengler V.H., Ivastinovic D., Haas A., Seidel G., Glats W., Malle E.M., Weger M., Velikay-Parel M. (2020). Gender differences in albumin and ascorbic acid in the vitreous antioxidant system. Free Radic. Biol Med..

[21] Lufrano D., Trejo S.A., Llovera R.E., Salgueiro M., Fernandez G., Damonte V.M., Flecha F.L.G., Raingo J., Ermácora M.R., Perelló M. (2016). Ghrelin binding to serum albumin and its biological impact. Mol. Cell. Endocrinol..

[22] Fasano M., Curry S., Terreno E., Galliano M., Fanali G., Narciso P., Notari S., Ascenzi P. (2005). The extraordinary ligand binding properties of human serum albumin. IUBMB Life.

[23] Addai-Mensah O., Gyamfi D., Duneeh R.V., Danquah K.O., Annani-Akollor M.E.E., Boateng L., Owiredu E.W., Amponsah F.A., Afrihie E.Y., Asare R. (2019). Determination of hematological reference ranges in healthy adults in three regions in Ghana. Biomed. Res Int..

[24] Indrayan A., Bhargava M., Shukla S. (2020). Reference einterval with age-gender variation for 4 liver function parameters in an adult segment of the Indian population. Int. J. Med. Biochem..

[25] Wilson A.M., Kimura E., Harada R.K., Nair N., Narasimhan B., Meng X.Y., Zhang F., Beck K.R., Olin J.W., Fung E.T. (2007). Beta2-microglobulin as a biomarker in peripheral arterial disease: Proteomic profiling and clinical studies. Circulation.

[26] Clement D.L. (2020). Hypertension and peripheral artery disease. J. Hypertens..

[27] Alende-casto V., Alonso-Sampedro M., Vazquez-Temprano N., Tuñez C., Rey D., Garía-Iglesias C., Sopeña B., Gude F., Gonzalez-Quintela A. (2019). Factors influencing erythrocyte sedimentation rate in adults: New evidence for an old test. Medicine.

[28] Mauvais-Jarvis F. (2018). Gender differences in glucose homeostasis and diabetes. Physiol. Behav..

[29] Homma H., Kurachi H., Nishio Y., Takeda T., Yamamoto T., Adachi K., Morishige K., Ohmichi M., Matsuzawa Y., Murata Y. (2000). Estrogen suppresses transcription of lipoprotein lipase gene. Existence of a unique estrogen response element on the lipoprotein lipase promoter. J. Biol. Chem..

[30] Price T.M., O’Brien S.N., Welter B.H., George R., Anandjiwala J., Kilgore M. (1998). Estrogen regulation of adipose tissue lipoprotein lipase-possible mechanism of body fat distribution. Am. J. Obstet. Gynecol..

[31] Pedersen S.B., Kristensen K., Hermann P.A., Katzenellenbogen J.A., Richelsen B. (2004). Estrogen controls lipolysis by up-regulating alpha2A-adrenergic receptors directly in human adipose tissue through the estrogen receptor alpha. Implications for the female fat distribution. J. Clin. Endocrinol. Metab..

[32] Pascot A., Lemieux I., Bergeron J., Tremblay A., Nadeau A., Prud’homme D., Couillard C., Lamarche B., Després J.P. (2002). HDL particle size:a marker of the gender difference in the metabolic risk profile. Atherosclerosis.

[33] Bredella M.A. (2017). Sex differences in body composition. Adv. Exp. Med. Biol..

[34] Deng W., Tan X., Zhou Q., Ai Z., Liu W., Chen W., Yu X., Yang Q. (2018). Gender-related differences in clinicopathological characteristics and renal outcomes of Chinese patients with IgA nephropathy. BMC Nephrol..

[35] Ramamani A., Aruldhas M.M., Govindarajulu P. (1999). Impact of testosterone and estradiol on region specificity of skeletal muscle-ATP, creatine phosphokinase and myokines in male and female Wistar rats. Acta Physiol. Cand..

[36] Yen C.H., Wang K.T., Lee P.P., Liu C.C., Hsieh Y.C., Kuo J.K., Bulwer B.E., Hung C.L., Chang S.C., Shih S.C. (2017). Gender-differences in the associations between circulating creatinine kinase, blood pressure, body mass and non-alcoholic fatty liver disease in asymptomatic asians. PLoS ONE.

[37] Skurtveit S., Tverdal A. (2022). Sex differences in gamma-glutamyl transferase in people aged 40-42 years in two Norwegian countries. Drug Alcohol Depend..

[38] Krishnamurthy H.A. (2013). The serum gamma glutamyl transpeptidase-A noninvasive diagnostic bio marker of chronic anicteric nonalcoholic liver disease. J. Clin. Diagn. Res..

[39] Rushton D.H., Barth J.H. (2010). What is the evidence for gender differences in ferritin and haemoglobin?. Crit. Rev. Oncol. Hematol..

[40] Arbiol-Roca A., Imperiali C.E., Montserrat M.M., Cerro A.S., Bosch de Basea A.C., Navarro L.S., Batch D.D., Politi J.V. (2018). Reference intervals for a complete blood count on an automated haematology analyser Sysmex XN in healthy adults from the southern metropolitan area of Barcelona. EJIFCC.

[41] Shaheen N.A., Rehan H., Moghairi A., Gmati G., Damlaj M., Salama H., Rather M., Mendoza M.A., Alanazi A., Al Ahmari B. (2022). Hematological indices in the adult Saudi population: Reference intervals by gender, age, and region. Front. Med..

[42] Grau M., Cremer J.M., Schmeichel S., Kunkel M., Bloch W. (2018). Comparisons of blood parameters, red blood cell deformability and circulating nutric oxide between males and female considering hormonal contraception: A longitudinal gender study. Front. Physiol..

[43] Strich D., Karavani G., Edri S., Chay C., Gillis D. (2017). FT3 is higher in males than in females and decreases over the lifespan. Endocr. Pract..

[44] Miyashita K., Murakami M., Iriuchijima T., Takeuchi T., Mori M. (1995). Regulation of rat liver type 1 iodothyronine deiodinase mRNA levels by testosterone. Mol. Cell Endocrinol..

[45] Suzuki S., Nischio S., Takeda T., Komatsu M. (2012). Gender-specific regulation of response to thyroid hormone in aging. Throid. Res..

[46] Stárka L., Pospisilová H., Hill M. (2009). Free testosterone and free dihydrotestosterone throughout the life span of men. J. Steroid. Biochem. Mol. Biol..

[47] Fabbrini E., Sullivan S., Klein S. (2010). Obesity and nonalcoholic fatty liver disease: Biochemical, metabolic, and clinical implications. Hepatology.

[48] Burra P., Bizzaro D., Gonta A., Shalaby S., Gambato M., Morelli M.C., Trapani S., Floreani A., Marra F., Brunetto M.R. (2021). Clinical impact of sexual dimorphism in non-alcoholic fatty liver disease (NAFLD) and non-alcoholic steatohepatitis (NASH). Liver Int..

[49] Chang Y., Jung H.S., Yun K.E., Cho J., Ahn J., Chung E.C., Shin H., Ryu S. (2016). Metabolically healthy obesity is associated with an increased risk of diabetes independently of nonalcoholic fatty liver disease. Obesity.

[50] Zeng S.M., Yankowitz J., Widness J.A., Strauss R.G. (2001). Etiology of differences in hematocrit between males and females: Sequence-based polymorphism in erythropoietin and its receptor. J. Gend. Specif. Med..

[51] Meffert C., Gerdes N. (2010). Program adherence and effectiveness of a commercial nutrition program: The metabolic balance study. J. Nutr. Metab..

[52] Lonardo A., Mantovani A., Lugari S., Targher G. (2020). Epidemiology and pathophysiology of the association between NAFLD and metabolically healthy or metabolically unhealthy obesity. Ann. Hepatol..

[53] Schiavo L., Pierro R., Asteria C., Calabrese P., Di Biasio A., Coluzzi I., Severino L., Giovanelli A., Pilone V., Silecchia G. (2022). Low-calorie ketogenic diet with continuous positive airway pressure to alleviate severe obstructive sleep apnea syndrome in patients with obesity scheduled for bariatric/metabolic surgery: A pilot, prospective, randomized multicenter comparative study. Obes. Surg..

[54] Ringel M.M., Ditto P.H. (2019). The moralization of obesity. Soc. Sci. Med..

