# Supplementary material for: Gender and Liver Steatosis Discriminate Different Physiological Patterns in Obese Patients Undergoing Bariatric Surgery: Obesity Center Cohort
Source: Nutrients. 2023 May 19;15(10):2381. doi: 10.3390/nu15102381 (PMC10221192; doi:10.3390/nu15102381)
Supplement: Supplementary file 1 [file nutrients-15-02381-s001.zip › Supplementary_Material.pdf]

nutrients

MDPI

Supplementary Materials

Table S1. Daily intake nutrients in different dietary regimen administered to the patient after to bariatric surgery.

Nutrients	Diets		
	Ketogenic	Low Carb	Hypocaloric
Protein (g)	74.4	89.44	62.8
Fat (g)	40.5	46.03	38.27
Carbohydrate (g)	32.8	97.56	165.53
Amid (g)	15.8	52.69	99.79
Total Fiber (g)	26	20.07	21.13
Cholesterol (mg)	0	209.55	128.3
Saturated Fat (g)	9.3	17.12	12.17
Polyunsaturated Fat (g)	1.74	6.68	4.16
Monounsaturated Fat (g)	14.66	32.97	18.94
Calcium (mg)	255.5	631.41	816
Sodium (mg)	85.93	529.15	733.56
Potassium (mg)	3609.4	4064.38	3112.03
Phosphorus (mg)	197.5	1287.27	1068.16
Iron (mg)	3.72	12.74	10.27
Zinc (mg)	3.91	18.8	6.87
Folic Acid (µg)	777	407.31	370.76
Niacin (mg)	6.43	18.7	17.04
Riboflavin (mg)	2.9	1.46	1.67
Thiamin (mg)	2.3	1.07	0.88
Vitamin 'A' (µg)	538.3	890.06	1316.49
Vitamin 'B6' (mg)	3.5	2.16	1.35
Vitamin 'C' (mg)	219.2	232.81	283.13
Vitamin 'D' (µg)	0	6.81	9.02
Vitamin 'E' (mg)	25	10.41	9.95
Insoluble Fiber (g)	10.26	13.63	11.69
Soluble Fiber (g)	2.88	2.68	4.34
Magnesium (mg)	521.7	241.91	223.88
Copper (mg)	0.63	1.84	1.05
Selenium (µg)	84.9	77.96	28.48
C4:0-C10:0 (g)	0	1.85	0.25
C12:0 Lauric (g)	0	5.81	0.47
C14:0 Myristic (g)	0	0.72	1.6
C16:0 Palmitic (g)	2.13	3.72	6.59
C18:0 Stearic (g)	0.61	1.17	2.1

(continue)

Nutrients	Diets		
	Ketogenic	Low Carb	Hypocaloric
C20:0 Arachidic (g)	0.15	0.22	0.16
C22:0 Behenic (g)	0	0	0
C14:1 Myristoleic Acid (g)	0	0	0.12
C16:1 Palmitoleic Acid (g)	0.16	0.36	0.42
C18:1 Oleic (g)	14.39	20.17	18.04
C20:1 Eicosaenoic (g)	0.06	0.11	0.06
C22:1 Erucic (g)	0	0	0
C18:2 Linoleic (g)	1.51	4.64	3.12
C18:3 Linolenic (g)	0.29	0.73	0.73
C20:4 Arachidonic (g)	0	0.02	0.06
C20:5 EPA (g)	0	0.06	0
C22:6 DHA (g)	0	0.16	0
Phytic Acid (g)	0	0.27	0.27
Lysine (mg)	239.66	7178.8	4224.59
Histidine (mg)	1836.83	5147.77	1756.57
Arginine (mg)	248.34	5357.77	3077.92
Aspartic Acid (mg)	531.05	8249.02	5129.28
Threonine (mg)	212.07	3758.27	2626.04
Serine (mg)	230.51	3790.4	2860.65
Glutamic Acid (mg)	1015.79	14262.13	12582.09
Proline (mg)	235.2	4021.22	4032.32
Glycine (mg)	272	4208.99	2280.89
Alanine (mg)	276.98	5066.8	3064.9
Cysteine (mg)	57.43	1014.03	1035.29
Valine (mg)	242.61	4219.87	3415.62
Methionine (mg)	73.2	2339.82	1377.51
Isoleucine (mg)	175.32	3721.07	2870.24
Leucine (mg)	337.87	6867.78	5213.72
Tyrosine (mg)	180.1	3007.68	2056.53
Phenylalanine (mg)	225.04	3793.29	2731.79
Tryptophan (mg)	64.91	891.43	689.14
Asparagine (mg)	0	0	0

(continue)

Nutrients	Diets		
	Ketogenic	Low Carb	Hypocaloric
Glutamine (mg)	0	0	0
Chlorine (mg)	0	0	0
Chromium (µg)	0.02	0	0
Fluorine (mg)	153.81	51.44	140.77
Iodine (µg)	182	216.52	41.93
Manganese (mg)	1.76	16.84	17.54
Molybdenum (µg)	0	12.1	11.78
Beta-Carotene (mg)	679.8	582.45	909.48
Alpha-Tocopherol (mg)	0	1.35	0.23
Vitamin K (µg)	0	6.82	7.26
Vitamin B5 (mg)	1.22	2.53	2.03
Vitamin B8 - Biotin (µg)	0	8.42	14.75
Vitamin B12 (µg)	3.75	7.31	1.03
Total Polyphenols (mg)	556.2	551.4	723.8
